# Harnessing the Fifth Element of Distributional Statistics for Psychological Science: A Practical Primer and Shiny App for Measures of Statistical Inequality and Concentration

**DOI:** 10.3389/fpsyg.2021.716164

**Published:** 2021-08-20

**Authors:** Ulrich S. Tran, Taric Lallai, Marton Gyimesi, Josef Baliko, Dariga Ramazanova, Martin Voracek

**Affiliations:** Department of Cognition, Emotion, and Methods in Psychology, School of Psychology, University of Vienna, Vienna, Austria

**Keywords:** statistical inequality, Lorenz curve, Gini index, entropy, interactive web application, R shiny, data visualization, statistics education

## Abstract

Although distributional inequality and concentration are important statistical concepts in many research fields (including economics, political and social science, information theory, and biology and ecology), they rarely are considered in psychological science. This practical primer familiarizes with the concepts of statistical inequality and concentration and presents an overview of more than a dozen useful, popular measures of inequality (including the Gini, Hoover, Rosenbluth, Herfindahl-Hirschman, Simpson, Shannon, generalized entropy, and Atkinson indices, and tail ratios). Additionally, an interactive web application (R Shiny) for calculating and visualizing these measures, with downloadable output, is described. This companion Shiny app provides brief introductory vignettes to this suite of measures, along with easy-to-understand user guidance. The Shiny app can readily be used as an intuitively accessible, interactive learning and demonstration environment for teaching and exploring these methods. We provide various examples for the application of measures of inequality and concentration in psychological science and discuss venues for further development.

## Introduction

A basic goal of quantitative empirical research lies in describing the properties of distributions, which arise from the collection of data, be these observational or experimental. In univariate descriptive statistics, there are four well-known central statistical moments: (1) the arithmetic mean (or expected value), (2) variance (and its square root, standard deviation), (3) skewness, and (4) kurtosis (with the latter two being so-called normalized or standardized moments, as their calculation also involves the second central moment). The arithmetic mean is informative about the central tendency (or location) of the distribution, variance of its spread (or dispersion), skewness of its symmetry, and kurtosis of its shape (i.e., tail extremity). Especially the first and second central moments are ubiquitously utilized in psychological science. In fact, their use is common to such an extent that reporting alternative descriptive distributional statistics, although these principally serve similar purposes (like, for example, the median, mode, midrange, trimmed or winsorized or weighted mean, or the harmonic or geometric mean, as measures of location; or the range, interquartile or semi-interquartile range, or mean or median absolute deviation, or the coefficients of variation or stabilization, as measures of dispersion), typically demand some extra justification and explanation for their use (the most recent edition of the *Publication Manual of the American Psychological Association*, 2020, p. 81, merely lists “the mean and standard deviation, and other measures that characterize data used” in its reporting standards for quantitative research designs). In contrast, the third and fourth central moments are much less often used, but still represent well-known statistical concepts within the field of psychological science and beyond.

Here, with this practical primer we intend to introduce to psychological science, and make more broadly known, a “fifth element,” or distributional aspect, from univariate descriptive statistics, namely, the family of statistical measures of inequality and concentration (for simplicity, hereafter referred to as inequality measures, as this is the umbrella term; Coulter, 1989). Measures of inequality have been developed since the early 20th century (for a historical review, see Piesch, 1975) and are important in many fields of research, including economics, political science and the social sciences, information theory, and biodiversity research in biology and ecology, to name but a few (Münzner, 1963; Bruckmann, 1969; Pielou, 1969; Allison, 1981; Magurran, 1988; Krämer, 1998). Yet, up to now, measures of inequality evidently are little-known, and certainly less used, in psychological science. Although in most cases the computation of statistical inequality measures is straightforward, as their closed-form formulas involve no estimations or iterations, attempts to transfer inequality measures into the behavioral and psychological sciences have been scarce. Typically, statistical textbooks for economists contain a chapter devoted to inequality measures (e.g., Krämer, 1998; Bleymüller et al., 2020), whereas introductory textbooks of descriptive and applied statistics written for psychological, sociological, or educational researchers mostly do not mention this topic at all (for notable exceptions, see, for instance, Polasek, 1994; Assenmacher, 2010).

Well-known applications of inequality measures concern monetary goods, such as income or capital, for individuals or households (e.g., OECD, 2011; Cobham and Sumner, 2014); votes in elections or monopolies in markets for companies and political parties (e.g., Wagschal, 1999; Fedderke and Szalontai, 2009); and the diversity of groups in sociology or species in biology (e.g., Hill, 1973; Jost, 2006). Wherever a good or unit (with “unit” being the preferred term hereafter; encompassing any unit of measurement with a lower bound of 0) is distributed among individuals, groups, or even whole countries (all of these invariably termed “components” hereafter; i.e., the objects, carriers, etc., to which the variable values pertain, see below), inequality may emerge (Coulter, 1989). Inequality is present whenever there is no equal distribution of units across components. Units may then be concentrated in only one or across a few components. Measures of inequality (in a narrower sense) quantify the extent to which the distribution of units over components is not equal, whereas measures of concentration are more concerned with the precise location of units and quantify whether units are concentrated in a few components or dispersed among numerous components (see Marfels, 1971; Coulter, 1989).

In psychological science, units could refer to scale and test scores^1^ or reaction times (in ms) in the case of metric variables, instead of capital or income. In the case of unordered categorical (i.e., nominal-scaled) or ordered categorical (i.e., ordinal-scaled) variables, units could refer to counts of diagnoses or school grades (i.e., regarding their prevalence), instead of votes (if diagnoses are counted within persons, this would render them a metric variable). In applications typical for the field of psychological science, components most often represent individuals (for metric variables) or groups of persons (for categorical variables), instead of countries, species, or political parties.

Measures of inequality are uniquely concerned with the distribution of units over the components. This type of distribution (see section The Lorenz Curve: A Probability Plot of the Unit Distribution for its probability plot) contrasts with the common frequency distribution, which displays the relative frequency (on the *y*-axis) of each value (on the *x*-axis; with usually binned values for metric variables). The unit distribution (as it is termed hereafter) displays the unit share (on the *y*-axis) of each component (on the *x*-axis; in ascending order of units). For categorical variables, the unit distribution is identical to the frequency distribution (with values sorted in ascending frequency). Here, the units are the counts, or frequencies, of the variable values themselves (i.e., the components). Measures of inequality thus provide alternative methods to describe the frequency distributions of categorical variables. For metric variables, the unit distribution differs notably from the frequency distribution. Here, measures of inequality offer unique insights into the data, which cannot be gained from the ordinary frequency distribution itself.

Applied to psychological science, inequality measures could therefore prove interesting and informative. Reaction-time data collected in experimental research, distributions and profiles of abilities and traits encountered in differential and personality psychology and in psychological assessment, or the diversity and comorbidity of disorders, as investigated in clinical psychology, can all be evaluated with measures of inequality, both within and between subjects or samples. Inequality statistics can also be used to detect influential or outlying cases (because outliers make up conspicuously high or low numbers of units) and to quantify aspects and effects not adequately represented by means and standard deviations, or frequencies alone (see Whelan, 2008, for an application to reaction-time data).

All the measures covered in this primer break down unit distributions into a single value to quantify inequality. However, measures differ (a) in the way they do this; (b) in the admissible scale levels for the variables investigated (unordered categorical vs. ordered categorical vs. metric); and (c) regarding their scope of application. (d) Most measures evaluate the whole unit distribution; however, there also are measures which exclusively scrutinize the distributional tails. Further, inequality may be quantified in (e) absolute or in relative terms. Absolute inequality arises when a large share of units is distributed among an only small *absolute* number of components; in contrast, relative inequality arises when a large share of units is distributed among an only small *relative* number of components (Bleymüller et al., 2020). For example, distribution *A* = (360, 250, 150) statistically is more unequal than distribution *B* = (180, 180, 125, 125, 75, 75) in absolute terms (as the units are distributed among fewer components for *A* than for *B*), but both distributions are commensurably unequal in relative terms (because the value sums, or units, of 360, 250, and 150 are distributed across one third of components each in *A* as well as in *B*). (f) Further, measures can be bounded (in most cases, limited to the range of 0–1) or unbounded. And (g), depending on how their minimum and maximum are defined, measures can either express distributional inequality or equality. The direction (or polarity) of any bounded measure can readily be reversed by subtracting the respective value from its maximum.

This practical primer introduces useful, popular measures of inequality to psychological science and presents a Shiny app (https://psychology-vienna.shinyapps.io/visualizing_inequality/; source code available on https://github.com/guitaric/Visualizing-Inequality) for their calculation and visualization, with downloadable output. Covered are (1) the Gini index, the Hoover index, and the Rosenbluth index; (2) the Herfindahl-Hirschman index and the Simpson index, alongside its associated measures Gini-Simpson index and inverse Simpson index; (3) Shannon entropy and the generalized entropy index, including the Theil index; and (4) the Atkinson index. This ensemble of measures is based on four different mathematical models (namely, in above order of (1)–(4), the deviations model, the combinatorics model, the entropy model, and the social welfare model; Coulter, 1989), the outlines of which are explained below. Since these distinct mathematical foundations constitute a straightforward and useful way for categorizing and understanding the variety of statistical inequality measures, our primer and its accompanying Shiny app (including the definitions and calculation formulas assembled in Table 1) are structured along this four-part scheme. (5) In addition, this primer covers distributional tail ratios; however, these further related approaches are not based on any of these four models and thus constitute a class of their own (for background and overview, see Voracek et al., 2013).

**Table 1:** Overview of the statistical inequality measures covered in this practical primer and implemented in the companion Shiny app.

**Model/Measure**	**Polarity**	**Admissible scale levels**	**Lower, upper limit**	**Formula**	**Synonyms**	**Interpretation**
**Deviations model**						
Gini index	Inequality	Categorical, metric	0, $1-\frac{1}{k}$	$G=\frac{1}{2k}\sum_{i=1}^{k}\sum_{j=1}^{k}|p_{i}-p_{j}|$		Area between the line of equality and the Lorenz curve
Corrected Gini index	Inequality	Categorical, metric	0, 1	$G^{\prime }=\frac{G}{1-\frac{1}{k}}$	*G* corrected for the number of components
Hoover index	Inequality	Categorical, metric	0, $1-\frac{1}{k}$	$H=\frac{1}{2}\sum_{i=1}^{k}|p_{i}-\frac{1}{k}|$	Pietra index (Pietra, 1948); Robin-Hood index (Atkinson and Micklewright, 1992); Schutz index (Schutz, 1951)	Greatest vertical distance between the line of equality and the Lorenz curve
Corrected Hoover index	Inequality	Categorical, metric	0, 1	$H^{\prime }=\frac{H}{1-\frac{1}{k}}$		*H* corrected for the number of components
Rosenbluth index	Inequality	Categorical, metric	$\frac{1}{k}$, 1	$R=\frac{1}{2\left(\sum_{i=1}^{k}ip_{i}\right)-1}$	Hall and Tideman index (Hall and Tideman, 1967)	Reciprocal value of twice the area above the concentration curve
**Combinatorics model**						
Herfindahl-Hirschman index	Inequality	Categorical, metric	$\frac{1}{k}$, 1	$HHI=\sum_{i=1}^{k}p_{i}^{2}$	Herfindahl index (Herfindahl, 1950), Hirschman index (Hirschman, 1945)	Probability that two random units stem from the same component
Simpson index	Inequality	Categorical	0, 1	$S=\frac{\sum_{i=1}^{k}n_{i}\left(n_{i}-1\right)}{N\left(N-1\right)}$	Simpson's *D* (diversity index; Magurran, 1988)	Probability that two random units stem from the same component
Gini-Simpson index	Equality	Categorical	0, 1	*GS* = 1−*S*	Blau index (Blau, 1977); Hunter-Gaston discriminatory index (Hunter and Gaston, 1988); Gibbs-Martin index (Gibbs and Martin, 1962); probability of interspecific encounter (PIE; Hurlbert, 1971)	Probability that two random units stem from different components
Inverse Simpson index	Equality	Categorical	1, inf	$IS=\frac{1}{S}$		Reciprocal value of the probability that two random units stem from the same component
**Entropy model**						
Shannon index	Equality	Categorical	0, log*_*a*_*(*k*)	$SI=-\sum_{i=1}^{k}p_{i}\text{lo}{\text{g}}_{a}p_{i}$	Shannon-Weaver index, Shannon-Wiener index (Spellerberg and Fedor, 2003)	Average information content of the unit distribution
Generalized entropy index	Inequality	Metric	0, ∞	$GE\left(\;\alpha \;\right)=\{\begin{array}{c} \frac{1}{k\;\alpha \;\left(\;\alpha \;-1\right)}\sum_{i=1}^{k}\left[{\left(kp_{i}\right)}^{\;\alpha \;}-1\right],\alpha \neq 0,\;1\\ \frac{1}{k}\sum_{i=1}^{k}kp_{i}\ln\;\left(kp_{i}\right)\;,\alpha =1\\ -\frac{1}{k}\sum_{i=1}^{k}\ln\;\left(kp_{i}\right)\;,\;\alpha =0 \end{array}$		Average redundancy in the unit distribution (with fine-tuning parameter α)
**Social welfare model**						
Atkinson index	Inequality	Metric	0, 1	$\begin{array}{l} AI\left(\;\varepsilon \;\right)=\\ \{\begin{array}{cc} 0, & \varepsilon =0\\ 1-\frac{k}{N}{\left(\frac{1}{k}\sum_{i=1}^{k}n_{i}^{1-\;\varepsilon \;}\right)}^{1/\left(1-\;\varepsilon \;\right)}, & 0<\varepsilon \neq 1\\ 1-\frac{k}{N}{\left(\prod_{i=1}^{k}n_{i}\right)}^{1/k}, & \varepsilon =1 \end{array} \end{array}$		Fraction of the total unit sum needed to attain a common unit standard for all components (with fine-tuning parameter ε)
**Tail ratios**						
Palma ratio	Inequality	Metric	$\frac{1}{4}$, ∞	$PR=\frac{\sum_{i=91}^{100}P_{i}}{\sum_{i=1}^{40}P_{i}}$		Ratio of the unit shares of the top 10% to the bottom 40% of the unit distribution
S80:S20 ratio	Inequality	Metric	1, ∞	$\text{S}80:\text{S}20=\frac{\sum_{i=81}^{100}P_{i}}{\sum_{i=1}^{20}P_{i}}$	20:20 ratio, quintile share ratio	Ratio of the unit shares of the top 20% to the bottom 20% of the unit distribution
P90:P10 ratio	Inequality	Metric	1, ∞	$\text{P}90:\text{P}10=\frac{P_{90}}{P_{10}}$		Ratio of the unit shares of the 90th percentile to the 10th percentile of the unit distribution
P50:P10 ratio	Inequality	Metric	1, ∞	$\text{P}50:\text{P}10=\frac{P_{50}}{P_{10}}$		Ratio of the unit shares of the 50th percentile to the 10th percentile of the unit distribution

*k = number of components (categories for categorical variables); n_i_ = number of units of the ith component (counts for categorical variables); N = total number of units; p_i_ = n_i_/N, the unit share of the ith component; log_a_ = logarithm to the base of a; P_i_ = unit share of the ith percentile of the unit distribution*.

In general, measures were selected for their frequency of use and their relevance in diverse fields of research (e.g., Coulter, 1989; Wagschal, 1999; Assenmacher, 2010; Cowell, 2011; OECD, 2011; Bleymüller et al., 2020) on the one hand, and their amenability to visualization on the other hand (further related measures, not covered here, are found in the referenced sources). The Shiny app provides intuitively understandable visualizations of these measures, which can be interactively modified in the app to grasp their functionality. Apps which would bundle a multitude of measures of inequality currently are not widely available, let alone apps which would provide visualizations for educational purposes as well. This primer and its companion Shiny app are thus intended to serve the triple aim of calling attention to these methods and approaches, providing a convenient means for calculating these measures, and helping users to better understand and learn about statistical measures of inequality through interactive visualizations.

In the following sections, we provide brief descriptions of the Lorenz curve (a probability plot of the unit distribution) and the various measures of inequality, their underlying mathematical models, their scope of application, and their characteristics and constraints. We utilize a nomenclature and notation amenable to psychological science and also provide 17 concrete examples and suggestions for applying inequality measures in psychological science, consecutively numbered within curly brackets, {Ex. 1} to {Ex. 17}. The above-mentioned general suggestions of using inequality measures for the detection of influential cases and outliers {Ex. 1} and the quantification of distributional aspects of reaction-time data (see Whelan, 2008) {Ex. 2} already constitute the first two of these examples. Further concrete examples and suggestions for some of the other applications listed above are provided in the following. The aim is to supply readers general impressions as well as varied specific ideas of how to beneficially apply various inequality measures in their own research and with their own data. We then describe the Shiny app and its functions and demonstrate its capacity for data visualization. The closing section discusses venues for future research and further methodological developments.

## Statistical Measures of Inequality and Concentration

The following definitions are essential for a basic understanding of this domain of univariate descriptive distributional statistics. The term *unit share* refers to the relative share (i.e., percentage) of the total unit sum belonging to a specific component. In the case of metric variables (e.g., scale or test scores, reaction times), the unit share relates to the *units of each individual* (with persons being components here). In the case of unordered or ordered categorical variables (e.g., diagnoses, school grades), the unit share relates to the *counts of the values of the categorical variable* (with the counts being units here, and the components representing the values of the categorical variable). The unit share of the *i*th component is denoted by *p*_*i*_ = *n*_*i*_/*N*, where *n*_*i*_ is the number of units (or counts, for categorical variables) and *N* is the total unit sum (or sum of counts, respectively).^2^ The term *component share* refers to the relative share (again, percentage) of the total number (denoted by *k*) of components belonging to a specific component. This notation is applicable to categorical and metric variables alike and therefore allows using the same labels in the formulas, thus simplifying and unifying their presentation.

Table 1 provides an overview of the measures covered here. Some of these go under different names, depending on research fields and traditions or authors (see Hirschman, 1964). Therefore, for the sake of clarity and comparison, Table 1 also lists synonyms of these, as commonly encountered in the literature. The labels of the measures in the formulas may sometimes diverge from those preponderantly used in the extant literature, in order to match them with the unified denominations used here.

### The Lorenz Curve: A Probability Plot of the Unit Distribution

**Figure 1:**
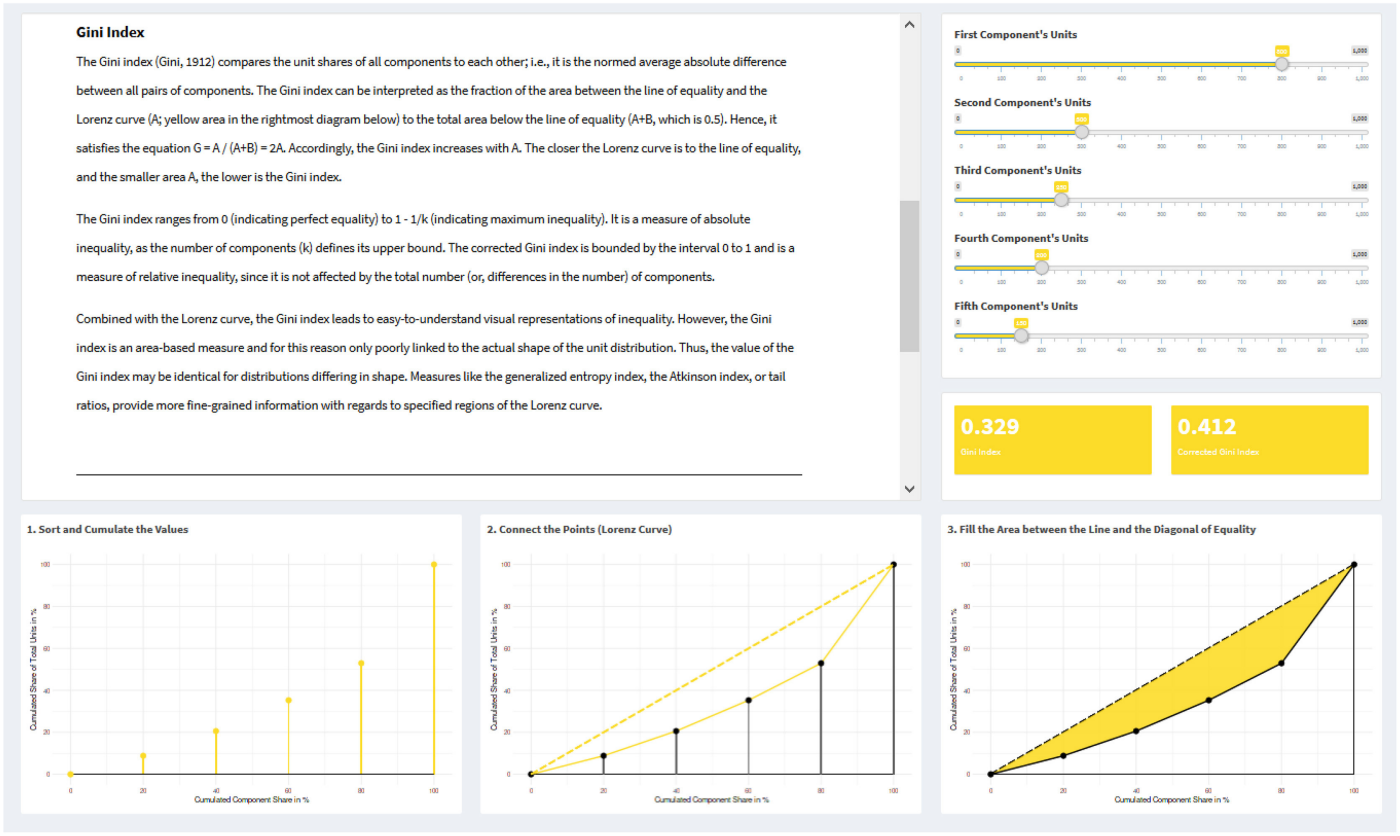
Example visualizations of the Lorenz curve and the Gini index from the interactive web application. On the upper left side, a text vignette explains the Gini index; on the upper right side, a slider panel with five sliders allows to create a distribution with five components; below this, a panel displays the value of the Gini index for the created distribution. At the bottom, three plots are created (based on the specific slider inputs), showing how a Lorenz curve is constructed and influenced by the data input, along with the area that corresponds to the Gini index.

The Lorenz curve (Lorenz, 1905) provides a straightforward visualization of the degree of statistical inequality (Gastwirth, 1972). It relates the unit share of to the component share (De Maio, 2007) and hence provides a straightforward visualization of the unit distribution. In essence, the Lorenz curve is a probability plot of the unit distribution, comparing it to a uniform distribution (see Figure 1, bottom).

The curve is drawn in a diagram, wherein the *x*-axis represents the cumulative percentage share of components and the *y*-axis the cumulative percentage share of units. Components are sorted in ascending order, beginning with the lowest unit share and ending with the highest one. The Lorenz curve is thus applicable to variables of any scale level (categorical or metric); however, for ordinal variables, their inherent ordering is ignored.

In the case that every component has the same share of units, the Lorenz curve manifests as the diagonal from the axis origin to the point (1, 1), or 100% of both axes, respectively. This diagonal is referred to as the *line of equality*. The sorting ensures that a Lorenz curve never exceeds this line of equality. The higher the inequality in a given distribution, the more the Lorenz curve will remain close to the *x*-axis, rising to the point (1, 1) and reaching it only from the far-right end of its possible range. In the extreme case of maximum inequality, where 100% of all the units fall into a single component, the Lorenz curve coincides with the *x*-axis over its total range, rising sharply to (1, 1) only at the rightmost position of its last component.

### Measures Based on the Deviations Model

Measures derived from the deviations model quantify inequality by considering the deviation of the components' unit share from a specific standard, as derived from the unit distribution itself. The Gini index and the Hoover index have direct links to the Lorenz curve (as does the Atkinson index, see section Measures Based on the Social Welfare Model: The Atkinson Index). Even though considered a measure based on the combinatorics model by Coulter (1989), the Rosenbluth index is also a measure derived from the deviations model, has intimate links to the Gini index, and thus is presented here. In psychological science, measures from the deviations model can be applied to quantify the inequality of the unit distribution of any metric variable, e.g., to investigate individual differences either within samples {Ex. 3} or between samples {Ex. 4}, or to detect outlying cases (or groups of outlying cases) {Ex. 1}. Yet, these measures can also be used to quantify inequality for categorical variables. For example, in epidemiology the Lorenz curve and the Gini index have been used to quantify differences in case rates between groups with different exposure risks (Lee, 1997) {Ex. 5} or seasonal variation phenomena in disease frequency (Lee, 1996) {Ex. 6}.

#### Gini Index

The Gini index (Gini, 1912) is the most widely known measure of inequality (Gastwirth, 1972; Ceriani and Verme, 2012). It is broadly used for evaluating income and wealth distributions of whole countries (OECD, 2011), but has also been applied in such diverse scientific fields as genetics, epidemiology, quality of life research, and engineering (e.g., Lee, 1996, 1997; Asada, 2005; Zonoobi et al., 2011; Jiang et al., 2016). Utilizing the Gini index, Asada (2005) quantified differences in health-related quality of life between individuals and groups of individuals {Ex. 7}.

The Gini index compares the unit shares of all components to each other; i.e., it is half of the normed average absolute difference between all pairs of components. Its modes of calculation are manifold: Gini (1912) already listed 13 calculation formulas (see Ceriani and Verme, 2012); the formula presented in Table 1 is yet another one. The Gini index can be interpreted graphically as the fraction of the area between the line of equality and the Lorenz curve (*A*) to the total area below the line of equality (*A* + *B*, which is 0.5). Hence, it satisfies the equation *G* = *A*/(*A* + *B*) = 2*A*. Accordingly, the Gini index increases with *A*. The closer the Lorenz curve is to the line of equality, and the smaller area *A*, the lower is the Gini index.

The Gini index ranges from 0 (indicating perfect equality) to 1–1/*k* (indicating maximum inequality). It is a measure of absolute inequality, as the number of components defines its upper bound. The corrected Gini index is bounded by the interval 0–1 and is a measure of relative inequality, since it is not affected by the total number (or, differences in the number) of components.

Combined with the Lorenz curve, the Gini index leads to easy-to-understand visual representations of inequality. However, the Gini index is an area-based measure and for this reason only poorly linked to the actual shape of the unit distribution. This implies that Gini index values may be identical for distributions differing in shape. In contrast, measures such as the generalized entropy index (section Generalized Entropy Index and Theil Index), the Atkinson index (section Measures Based on the Social Welfare Model: The Atkinson Index), or tail ratios (section Tail Ratios), provide more fine-grained information with regards to specified regions of the Lorenz curve.

#### Hoover Index

The Hoover index (see Hoover, 1936; but actually dating back at least to pre-World War I time and the Italian economist and statistician Constantino Bresciani-Turroni: see Kondor, 1971), is one of the simplest inequality measures. In other scientific fields, the Hoover index is used to quantify regional income inequality (Huang and Leung, 2009) or the spatial concentration of populations (Santic, 2014). It can be interpreted as the share of units that need to be redistributed across components to achieve perfect equality (or, couched in an economic context: the amount of money which must be taken from the rich and given to the poor to achieve an equal distribution; hence, its synonym “Robin-Hood index”).

The Hoover index compares the unit shares of all components to their overall mean; it is based on the sum of absolute deviations. Similar to the Gini index, the Hoover index can be visualized via the Lorenz curve and is a measure of absolute inequality in its unbounded form. Its value is equal to the maximum vertical distance between the Lorenz curve and the line of equality, thus ranging from 0 (for maximum equality, when the Lorenz curve coincides with the line of equality) to 1–1/*k* (for maximum inequality). The corrected Hoover index ranges from 0 to 1 and is a measure of relative inequality.

#### Rosenbluth Index

The Rosenbluth index (Rosenbluth, 1955) is used in economics to determine how strongly a market is dominated by a monopoly (e.g., Fedderke and Szalontai, 2009). For calculation, it draws on the rank order of components and compares each component's unit share to the probability that two units belong to the same component (Coulter, 1989). This approach makes it a measure likewise rooted in the deviations model as well as in the combinatorics model (see section Measures Based on the Entropy Model).

Because of its rank-order definition, the Rosenbluth index can be plotted in a diagram similar to the Lorenz curve. However, the components are sorted in descending order (not, as otherwise customary, in ascending order), such that the first entry in the diagram contains the highest (not the smallest) unit share. Also, the *x*-axis of the Rosenbluth index diagram does not cover the cumulative component shares in percent (1–100%), but rather the component numbers (1 to *k*) instead. For five components, the *x*-axis would range from 1 to 5, and the total area of the diagram would equal. The diagonal in this rectangle again indicates the line of equality.

Like the Gini index, the Rosenbluth index is an area measure. It is the reciprocal value of twice the area (*A*) above the concentration curve, *R* = 1/(2*A*). Thus, the Rosenbluth index can also be expressed in terms of the uncorrected Gini index (Marfels, 1971): *R* = 1/[*k*(1 − *G*)].^3^ In the case of maximum equality, the curve coincides with the line of equality, and the area above the curve is identical to the right-angled triangle above the line of equality,. Thus, the lower bound of the Rosenbluth index is 1/[2·(*k*/2)] = 1/*k*. In the case of maximum inequality, the curve rises immediately steeply from (0, 0) to (1, 1), and then runs horizontally to the rightmost point (*k*, 1). The resulting (minimum) area above the concentration curve is then defined by a right-angled triangle having the corner points (0, 0), (0, 1), and (1, 1), thus spanning a total area of (1·1)/2 = 0.5, in which case the Rosenbluth index yields a value of 1/(2·0.5) = 1.

### Measures Based on the Combinatorics Model

Measures derived from the combinatorics model quantify the probability that two randomly selected units belong to the same component (or, alternatively, to different ones). The Simpson index, the Gini-Simpson index, and the inverse Simpson index are appropriate for categorical variables. The Herfindahl-Hirschman index, in principle, can be applied to both categorical and metric variables (but see section Herfindahl-Hirschman Index). For large *N*, the indexes of Herfindahl-Hirschman and Simpson converge in value.

#### Herfindahl-Hirschman Index

In economics, the Herfindahl-Hirschman index (Hirschman, 1945, 1964; Herfindahl, 1950; HHI) is used to describe market concentrations and costumers' degree of brand loyalty, and in political science, the degree of fragmentation of political parties in elections (Wagschal, 1999).

The HHI is based on an urn model with replacement (i.e., drawing random units does not change the components' unit shares) and is calculated by summing the squared unit shares of every component of the unit distribution (Coulter, 1989). Larger unit shares are disproportionally weighted more heavily than smaller unit shares (e.g., 0.8^2^ = 0.64, but 0.1^2^ = 0.01). The HHI reaches its maximum value of 1, when a single component possesses the total share all on its own, and its minimum value of 1/*k* for evenly distributed shares. Components without a share, also called null components, are ignored in computing the HHI. Due to its sensitivity to the number of components, but its disregard of null components, the HHI represents a special case of an absolute inequality measure.

The HHI is suitable for reflecting market power because of its emphasis on large shares and its insensitivity for small shares. To illustrate, in a market with many companies which sell very small numbers of products, these companies would be of little or no importance for estimating the extent of market separation. In psychological science, the HHI can be used for analyzing group membership {Ex. 8} and market segregation {Ex. 9} in social and marketing psychological applications. If used for outlier detection {Ex. 1}, its negligence of small unit shares makes the HHI only sensitive for outliers at the high end (whereas not at the low end) of the unit distribution. For applications to categorical variables, *N* needs to be relatively large, as the urn model with replacement is only asymptotically correct under such circumstances. For small-*N* applications, indices from section Simpson Index, Gini-Simpson Index, and Inverse Simpson Index should be used instead.

#### Simpson Index, Gini-Simpson Index, and Inverse Simpson Index

The Simpson index (Simpson, 1949) is intended for categorical variables and mainly used for the quantification of biodiversity (Hill, 1973; Jost, 2006). For large *N*, the Simpson index and the Herfindahl-Hirschman index converge in value. However, numerical differences may be large for small *N*. The Simpson index then is the appropriate and correct measure.

The Simpson index is defined as the probability that two randomly selected units (e.g., individuals) belong to the same component (e.g., group), and its limits are 0 (0%) and 1 (100%). It is a measure of inequality and based on an urn model without replacement (i.e., drawing random units decreases the components' unit share). The complementary probability of the Simpson index (1–*S*), denominated as Gini-Simpson index, can be interpreted as the probability that two randomly selected units belong to different components. The inverse Simpson index (i.e., its reciprocal value, 1/*S*) maps the values of the Simpson index onto the interval [1, ∞], with positive infinity corresponding to a probability of 0 for the Simpson index and 1 to a probability of 1. The Gini-Simpson index and the inverse Simpson index are measures of equality.

For psychological science, the Simpson index and the Gini-Simpson index appear equally appealing, as they provide straightforwardly interpretable, dimensionless numerical information (namely, probabilities) to quantify diversity in categorical variables, such as diagnoses {Ex. 10}, sexual orientation {Ex. 11}, or discrete preferences and traits, like handedness (e.g., Tran et al., 2014) {Ex. 12}. All three indices are especially appealing for small-sample applications, under which conditions the usage of the HHI would be inappropriate (e.g., country of origin in systematic reviews and meta-analyses encompassing only a few, up to a couple of dozen, primary studies {Ex. 13}).

### Measures Based on the Entropy Model

Entropy is a measure for the degree of disarray, disorganization, or unpredictability in diverse physical systems (particularly, in thermodynamics) and for uncertainty in information theory. Its usage as a measure of inequality follows the idea that, in the case of categorical variables, components (e.g., diagnoses) occurring very frequently or very rarely have low information content by their nature, whereas components occurring with medium probability naturally carry much higher information content. Thus, discrete distributions with more components, or with components having more evenly distributed units, carry a higher total information content than distributions with less evenly distributed units or, at the extreme, only one component. The Shannon index is intended for categorical variables, whereas the generalized entropy index and the Theil index for metric variables.

#### Shannon Index

The Shannon index (Shannon, 1948; Shannon and Weaver, 1949) was originally devised to determine the information content of a message (e.g., different letters occurring in a text; Voracek et al., 2007 {Ex. 14}). However, it is also utilized for the examination of species diversity in ecosystems (Spellerberg and Fedor, 2003). The Shannon index relates the uncertainty of an event to the gain of the received information. A maximum of information is gained when the certainty of a given unit is minimal, namely those observed with a probability of 0.5 (or proportion of 50%) for dichotomous (yes vs. no) outcomes. The more certain the occurrence of the next unit (regardless of whether it is highly likely or highly unlikely), the lower the respective information content.

The quantity of bit (representing a logical state with a single value, out of two possible values) is common as the basic unit of information within information theory; hence, the binary logarithm (log2) is often used calculating the Shannon index. However, using other logarithmic bases is also feasible, such as the natural logarithm (ln) or the logarithm to the base of 10 (log10). For log2, the Shannon index can be interpreted as the average number of yes/no-questions that must be asked to determine to which component a randomly selected unit of the distribution belongs to. For example, a string consisting only of “A” s would result in a Shannon index of 0 because, trivially, every letter is an “A.” The event of having an “A” on every position is 100% sure. In contrast, the string “ABCD” with evenly distributed letters would result in a Shannon index of 2, because two questions would be sufficient to determine each letter (namely, the first question being: “AB or CD?”; and the second question being: “A or B?” or, equivalently, “C or D?”). Duplicating each letter (“AABBCCDD”) would result in the same Shannon index as for the string “ABCD.” Null entries are ignored. The Shannon index has a lower bound of 0 and an upper bound determined by the respective logarithm (whichever is used) of *k* (the number of components, i.e., categories).

#### Generalized Entropy Index and Theil Index

These measures (going back to Theil, 1967; for a derivation of the generalized entropy index, see Shorrocks, 1980) generalize the idea of entropy and compare the unit shares of each individual component to the mean of all components (see Footnote 2; *kp*_*i*_ could also be substituted by $n_{i}/\bar{n}$ in Table 1). They quantify the distance between the highest possible entropy (i.e., the uniform distribution of units over components) and the observed entropy. Thus, they measure inequality (in the sense of redundancy), rather than equality (such as the Shannon index).

The unique characteristic of the generic form of these measures, as represented by the generalized entropy index, lies in its parameter α. Setting α = 1 yields the Theil index (Theil, 1967), which is a direct generalization of the Shannon index for metric variables. Setting α = 0 yields the mean log deviation (i.e., the average of the deviations of the log units to their log mean; Theil, 1967). More generally, the value of α fine-tunes the generalized entropy index to specified ranges of the unit distribution with regards to the effects of hypothetical unit transfers from one component to another on its numerical value. That is, the more positive α, the more sensitive the index is to inequality at the high range of the distribution; conversely, the more negative α, the more sensitive it is to inequalities occurring at the low range of the distribution. Thus, the Theil index is more sensitive to inequalities at the high range of the distribution than the mean log deviation. The generalized entropy index thus provides a convenient way to differentially weigh inequality at (and, in a way, to “zoom into”) the high vs. low ranges of the unit distribution. Consequently, it allows for a more fine-grained inspection of inequality than merely area-based measures (such as the Gini index) are capable of.

### Measures Based on the Social Welfare Model: The Atkinson Index

The Atkinson index (Atkinson, 1970, 1987, 2008) is a measure of income inequality with intimate links to the generalized entropy index (and thus to the Lorenz curve as well). It is derived from the so-called social welfare model, which is specifically concerned with the effects of redistributing units from the high range of the unit distribution to its low range. Similar to the generalized entropy index (*GE*), the Atkinson index (*AI*) is characterized by an additional parameter, in this case ε (“inequality aversion”). Unlike α, ε is restricted to non-negative values. The two parameters are related through the equation ε = 1 − α, as are the indices themselves, namely, *AI* = [ε (ε −1)*GE*]^1/(1− ε)^, for ε ≠ 1, and *AI* = 1 − *e*^−*GE*^, for ε = 1. Atkinson (1970) recommended to set ε values between 1.5 and 2 (which results in α values between −0.5 and −1). Setting ε to 0 results in an Atkinson index of 0 for all distributions. Otherwise, the Atkinson index increases with increasing ε, given that the distribution is not perfectly equal. The parameter ε is a fine-tuning parameter like α, but allows to “zoom in” only into the low range of the distribution (i.e., it assigns more and more weight to redistribute units from the high range to the low range).

In its original conceptualization and application, the Atkinson index is meant to be interpreted in relation to a social welfare function and the “welfare equivalent equally distributed income,” *n*_ε_. This common standard depends on the value of the Atkinson index itself (and, hence, on the choice of ε) and is defined, in more general terms, as the average of units across all components multiplied by one minus the Atkinson index, $n_{\;\varepsilon \;}=\bar{n}\left(1-AI\right)$. The Atkinson index thus satisfies the equation $AI=1-n_{\;\varepsilon \;}/\bar{n}$. For ε = 0, the Atkinson index is 0 and the standard is equal to the average of units. For all other cases, the Atkinson index quantifies the fraction of units of the total unit sum, which is needed to reach this common standard for each component, if units were distributed equally. For example, with an Atkinson index of 0.4, the common standard could be reached for each component with only 60% () of the total unit sum, if units were distributed equally. In the case of an equal unit distribution, the Atkinson index is 0, and the unit share of every component already corresponds to the common standard.

The concept of the Atkinson index is rather abstract, with its value depending on a priori “accepted” levels of inequality (as expressed by ε). Yet, it is exactly this reference to a common standard which makes the Atkinson index interesting for psychological science as well. Reversing its common application, the parameter ε can be fine-tuned to yield a value for the common standard for given data that has a substantive interpretation: e.g., a threshold (in ms) for valid trials in experimental reaction-time data {Ex. 15}, or a clinically relevant cutoff for symptom checklists^4^ {Ex. 16}. The Atkinson index then quantifies inequality, and can be interpreted, with reference to this specified value. Via the link to the generalized entropy index, inequality can easily be expressed in an alternative metric as well.

### Tail Ratios

One of the simplest ways to measure inequality is to compare the shares of the two tail sections of the unit distribution exclusively, while ignoring its middle section. This provides measures of inequality which are specifically sensitive to any differences located in the distributional tails (e.g., Langel and Tillé, 2011; Voracek et al., 2013) and more informative of the shape of the unit distribution than mere measures of area, like the Gini index. Distributional tail ratios are intended for metric variables. When the number of components is small, they should be interpreted with caution, because of possible effects due to rounding errors (e.g., imagine estimating the richest 20% based on merely three components).

#### Palma Ratio, S80:S20, and Percentile Ratios

The Palma ratio (Palma, 2011) is widely used to assess gross national income distributions. It is the ratio of the unit shares of the top 10% to the bottom 40% of the unit distribution. Applied to the gross national income, the Palma ratio tends to vary between 0.8 and 1.8 across Europe (OECD, 2011). A value of 1.8 indicates that the top 10% possess 1.8 times the units that the bottom 40% do. The S80:S20 ratio compares the share of the top fifth with the share of the bottom fifth of the unit distribution. It follows the logic of the Pareto distribution (Pareto, 1896) and its associated Pareto principle (known as the 80-to-20 rule, or the principle of factor sparsity: for many real-world phenomena, about 80% of effects or consequences have been observed to be due to about 20% of the causes or sources). Percentile ratios compare selected percentiles of the unit distribution. The P90:P10 ratio compares the 90th percentile with the 10th percentile, whereas the P50:P10 relates the median (50th percentile) to the 10th percentile.

## Shiny App

### Visualizing Inequality Measures

The web application “Visualizing Inequality” was developed in the statistical computing environment R (R Core Team, 2018), utilizing the R shiny package (Chang et al., 2018), and has been tested with the web browsers Mozilla Firefox, Google Chrome, and Microsoft Edge. It can be accessed at https://psychology-vienna.shinyapps.io/visualizing_inequality/ (source code available on https://github.com/guitaric/Visualizing-Inequality). In this Shiny app, each measure has its own page and comes with its own built-in example. The app provides interactive plots, such that changes in the numerical value of an inequality index can not only be understood via accompanying text and tables, but, above all, visually and thus intuitively. All plots in the app have been created with the R package ggplot2 (Wickham, 2016). Inequality measures provided in the app have been validated against the numerical output of the respective functions implemented in the R packages REAT (Wieland, 2019), ineq (Zeileis and Kleiber, 2015), and diverse (Guevara et al., 2017), as well as cross-checked against further procedures provided in the biodiversity online calculator at https://www.alyoung.com/labs/biodiversity_calculator.html. All library dependencies relevant for the app are listed on https://github.com/guitaric/Visualizing-Inequality/blob/master/ui.R. A test file for cross-validation of the app with other R packages is available on https://github.com/guitaric/Visualizing-Inequality/blob/master/testfile.R.

**Figure 2:**
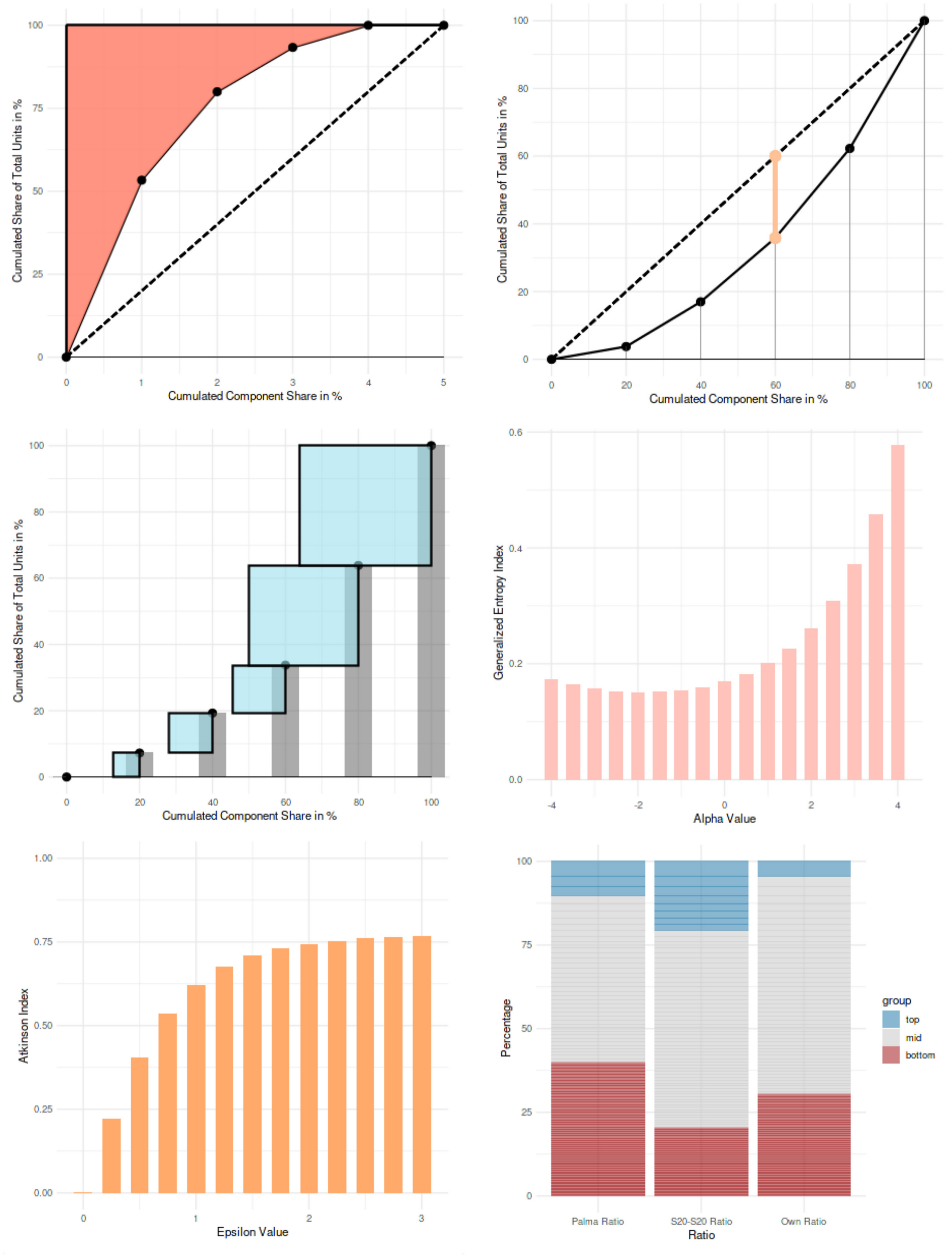
Example visualizations from the interactive web application (from left to right and from top to bottom): Rosenbluth index, Hoover index, Herfindahl-Hirschman index, generalized entropy index for different values of the fine-tuning parameter α, Atkinson index for different values of the fine-tuning parameter ε, and tail ratios.

The visualizations of the Gini index (Figure 1), the Rosenbluth index, the Hoover index, and the Herfindahl-Hirschman index (Figure 2) are all similarly designed. For each of these indices, there are five sliders which can be controlled individually. The sliders represent one component each and control their unit numbers. Upon changing a value, i.e., the unit number of a component, via a slider, the value of the corresponding measure as well as its visualization are updated.

On the page illustrating the Simpson index, five optional numerical inputs are implemented. Values from 0 to 10 can be entered to represent the occurrence of a component (e.g., a certain diagnosis). By entering the occurrence numbers, a plot shows the numbers for each component. Components are illustrated with color-coded symbols, and the plot is automatically updated whenever numbers are changed. The plot is not the actual visualization of the Simpson index; rather, it provides users with a visualization of the membership of every unit across the different components, to allow for a better grasp of the calculated probabilities. Whenever inputs are changed, the index is automatically recalculated and updated. The Simpson index, the Gini-Simpson index, and the inverse Simpson index are all shown adjacent to each other.

For the Shannon index, the information content of a message is presented. On this page, users can generate a string of letters, ranging from A to F, by clicking on the corresponding buttons. Buttons to delete the last letter, or the whole string, are provided. While building a string of letters, a constantly updated table lists the values to calculate the Shannon index. The first column shows the units (i.e., counts) of each component (i.e., the letters), the second column lists the unit shares of the components (i.e., their relative frequencies), the third column the logarithms of the unit shares (log2, ln, or log10), and the fourth column the negative products of the unit shares and their logarithms. Building their sum yields the Shannon index.

The generalized entropy index and the Atkinson index (Figure 2) are presented with predefined unit distributions, in order to demonstrate the behavior of these indices, conditional on the values of the parameters α and ε. For the generalized entropy index, α values range from −4 to 4 in the bar chart; for the Atkinson index, ε values range from 0 to 3.

For the tail ratios (Figure 2), real-world datasets are used for demonstration (taken from International Olympic Committee, 2018; World Health Organization, 2018; United Nations, 2019a,b; Government Digital Service United Kingdom, 2020; provided on https://github.com/guitaric/Visualizing-Inequality/tree/master/Data%20files). The application to country-level suicide rates (World Health Organization, 2018) is a further example for the application of inequality measures in psychological science {Ex. 17}. A stacked bar chart shows the unit shares of the top and bottom component shares for the Palma ratio, the S80:S20 ratio, and for a user-defined ratio (i.e., any other ratios can be calculated as well).

### Processing Datasets

**Figure 3:**
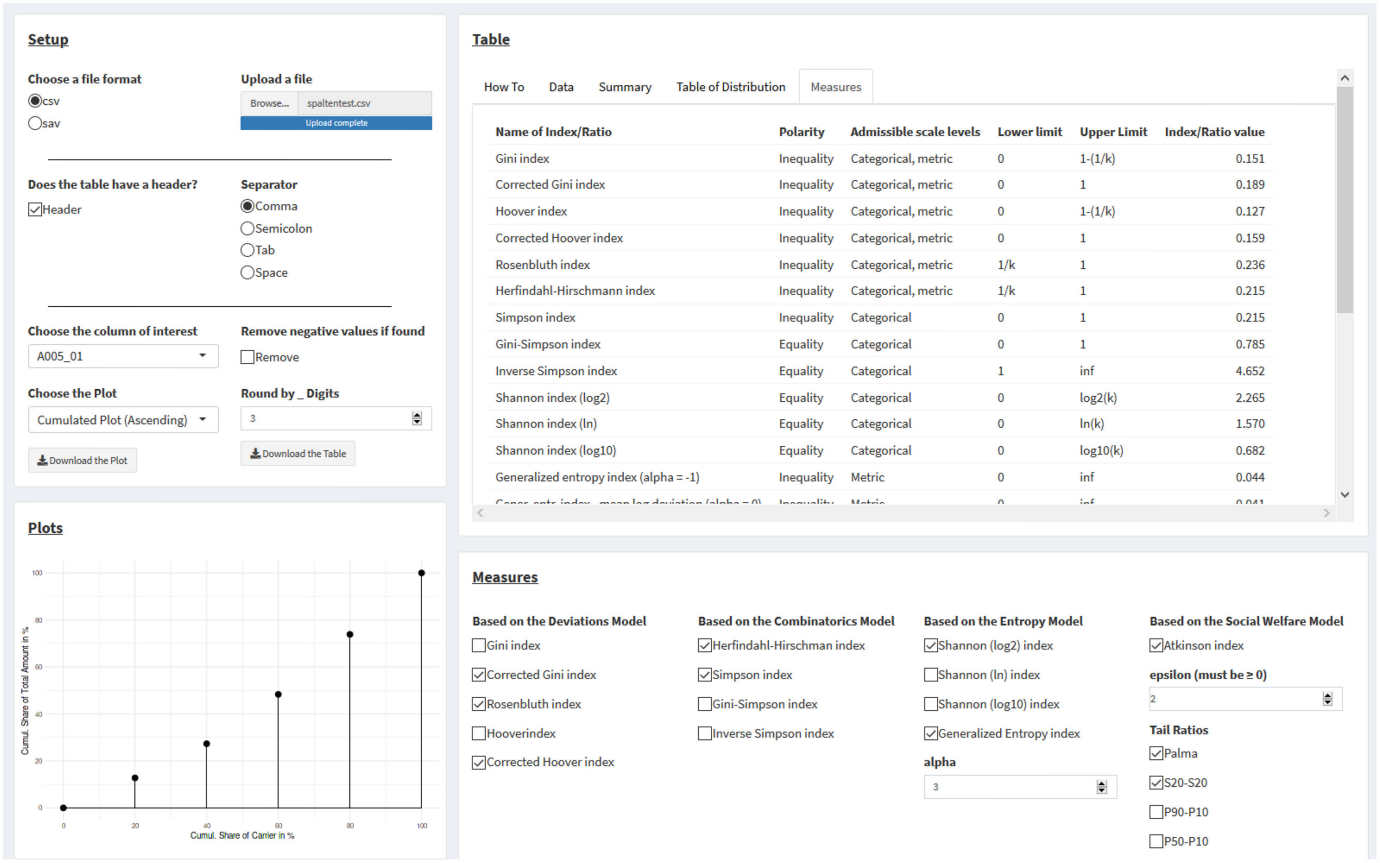
Calculating statistical measures of inequality from one's own datasets. See main text for details.

The page “Calculate” allows users to upload data for visualization and for calculating every index discussed here (Figure 3). On the left panel “Setup,” users can upload and set up data (up to a maximum file size of 5 MB). The tab “How to” in the “Tables” panel on the right side of the page gives instructions for use.

First, users must select the filetype, of which .csv (e.g., from MS Excel) and .sav (IBM SPSS) files presently are supported. For .csv files, the user must specify within the “Setup” panel whether there is a column header, along with the appropriate separator for data entries, if the data are not comma-separated. The tab “Data” in the panel “Tables” interactively displays the data under the current settings, so that users can make sure the data are correctly read in by the app.

After the upload, all dataset columns are listed and can be selected in a drop-down menu within the “Setup” panel on the right side of the page titled “Choose the columns of interest.” Data need to be in a numerical format; otherwise, a warning is displayed. Selecting a column with numerical data immediately results in processing this column's data. NA values are automatically ignored, but can be seen in the “Data” tab of the “Tables” panel on the right-hand side of the page. If negative values are present, a warning message will be printed in the “Plot” panel in the lower left corner of the page, where a user-selected plot would appear otherwise, and plotting and the calculation of measures only resumes by clicking the “Remove” checkbox.

Plots are displayed for numerical columns with exclusively non-negative values. Users can select between two cumulative plots (ascending and descending) and visualizations of the Gini index, the Rosenbluth index, the Hoover index, the Herfindahl-Hirschman index, the generalized entropy index, the Atkinson index, and of tail ratios. For visualization, components are merged into 20 aliquot parts (i.e., of 5% each), should their number exceed 20. However, all calculations are based on the actual (not on the merged) number of components.

On the panel “Measures” at the right lower part of the page, values for the selected measures in the checkbox list are displayed. For the generalized entropy index and the Atkinson index, numerical values for the α and ε parameters must be supplied.

Next to the “Data” tab on the upper right side, a “Summary” tab displays summary data for all variables, and a “Table of Distribution” tab displays a table for the selected variable with the following columns: values (sorted); share of units in %; cumulated share of units in %; share of components in %; and cumulated share of components in %. The rightmost tab in the row, labeled “Measures,” displays a table of all measures in the app, including their polarity, the admissible scale levels, the measures' respective lower and upper limits, and the actual calculated values of the inequality measures, based on the current data.

By clicking on the two download buttons on the “Setup” panel, the current plot can be downloaded as a .png file and the table with the values of the inequality measures as a .csv file.

## Discussion

This practical primer provides a concise, structured introduction to the domain of statistical inequality measures, mainly intended for applications in psychological science (assembling 17 concrete examples and suggestions for their use), and is accompanied by a web application for visualizing and calculating this suite of measures. The app provides a user-friendly way to process one's own data, to compute the discussed measures for variables of interest, and to visualize the unit distribution of these, which lies at the heart of many inequality measures. By highlighting areas of the unit distribution which are relevant to the measures of interest, the generated plots aid the user in developing an intuition for them. While the utilization of inequality measures may well be considered as “exotic” in psychological science, we have confidence that this primer and its companion Shiny app will help to foster and facilitate more widespread use of statistical inequality measures in this field. Alongside the commonly used descriptive distributional statistics, like the arithmetic mean and the standard deviation or variance in particular (and, less commonly used, distributional skewness and kurtosis), inequality (or concentration) measures, taken as the “fifth element” of univariate descriptive distributional statistics, can prove useful in the context of the communicative purposes related to a variety of research questions and findings in diverse subfields across psychological science.

While with this primer we intend to make measures of inequality better known within psychological science, there also remain a number of additional points and considerations which still may be fruitfully addressed in future research and updates of the Shiny app. First, for many inequality measures, their variance and standard error is known (even though there sometimes is disagreement, or confusion, about their correct calculation; see Langel and Tillé, 2013). For some inequality measures, this is also true for applications in complex sampling designs (e.g., Biewen and Jenkins, 2006; Langel and Tillé, 2013). Standard errors enable the calculation of confidence intervals and would therefore make inequality measures even more interesting from the view of analytic practices based on inferential statistics, as typically applied in psychological science. Bootstrap confidence intervals could be provided for measures for which calculating standard errors based on closed-form formulas currently is unknown or intractable, or when sample size is small.

Second, the Gini index, the generalized entropy index, the Atkinson index, and the measures based on the combinatorics model all allow for a convenient decomposition of distributional inequality across subgroups (e.g., Shorrocks, 1980; Lambert and Aronson, 1993; Lande, 1996). This analytic possibility naturally lends itself to manifold research questions, as addressed in psychological science. Implementing such additional functionality into the Shiny app would allow for even more fine-grained analytic options in various applications and would open up new venues of research.

Third, there are a number of statistical inequality measures, not adopted here, which could be incorporated into the Shiny app as well, such as Wilcox's deviations from the mode (Wilcox, 1973), or Lieberson's index of diversity (Lieberson, 1969). Further examples include the well-known coefficient of variation (also denominated as the relative standard deviation), suited for metric (ratio-scaled) variables and defined as the ratio of the standard deviation to the (absolute value of the) mean, along with its little-known reciprocal value (the coefficient of stabilization). Although widely used as descriptive, standardized measures of the dispersion of distributions (for instance, in fields like endocrinology or analytical chemistry, to quantify assay precision and reliability), it is lesser known that the coefficients of variation or stabilization can as well be interpreted as measures of statistical concentration (Liu and Zheng, 1989).

In essence, the present selection of measures of inequality is meant as representative, not exhaustive, and was guided by the importance and utilization of the respective measures in the extant scholarly literature across various disciplines, and a preference for variety of the selected measures with respect to the mathematical models from which they are deduced. Further measures could easily be incorporated in future updates of the Shiny app.

Apart from providing a convenient tool to visualize and calculate measures of statistical inequality for one's own datasets, the Shiny app provides an overview of all the measures discussed here at one glance and in a structured way, along with providing clues regarding their interpretation and suggestions for meaningful application. In this way, the Shiny app may also serve as an intuitively accessible learning and demonstration environment in the context of exploring these methods and teaching them in psychology and elsewhere. The Shiny app is scheduled to be updated regularly in the future. Measures and functions not yet included (e.g., alternative file types for input data, or extensions to the set of the 16 different measures currently offered) are envisaged to become available with forthcoming versions.

## Data Availability Statement

Publicly available datasets were analyzed in this study. This data can be found at: https://psychology-vienna.shinyapps.io/visualizing_inequality/.

## Author Contributions

UT supervised the project, provided critical feedback for the creation of the Shiny app, and wrote the draft of the manuscript. TL created the Shiny app and contributed to the draft of the manuscript. MG, JB, and DR provided critical feedback on the Shiny app and on the draft of the manuscript. MV conceived the original research idea, co-supervised the project, and contributed to the draft of the manuscript. All authors read and approved the final manuscript and agree to be accountable for the content of the work.

## Conflict of Interest

The authors declare that the research was conducted in the absence of any commercial or financial relationships that could be construed as a potential conflict of interest.

## Publisher's Note

All claims expressed in this article are solely those of the authors and do not necessarily represent those of their affiliated organizations, or those of the publisher, the editors and the reviewers. Any product that may be evaluated in this article, or claim that may be made by its manufacturer, is not guaranteed or endorsed by the publisher.

## Footnotes

^1^Scores need to have a lower bound of 0, such that they have well-defined sums when added together. Ratio scales satisfy this requirement by definition. For any interval-scaled variables, this requirement may be satisfied with suitable linear transformations (location shift), should negative values occur. While this type of variable transformation does not change the scale type (i.e., measurement level or structure), it makes scores amenable to the evaluation of inequality. For further discussion regarding the adequacy of interval scales for measuring inequality, see Allison (1978).

^2^For metric variables, unit shares are just the linearly transformed (scale-shifted) original values. Yet, one needs to remember here that *k* refers to the number of individuals and *N* (uncommonly so) to the total sum of their original values. Further notice that in this case the unit share *p*_*i*_ implicitly relates *n*_*i*_ to its mean (i.e., $\bar{n}=N/k$): $N=k\bar{n}$, and therefore, $p_{i}=n_{i}/k\bar{n}$ and $kp_{i}=n_{i}/\bar{n}$.

^3^As the total area in the Rosenbluth diagram is *k* (not unity, as in the diagram of the Lorenz curve), the factor *k* also appears in this formula.

^4^If this common standard is greater than the sample mean, the polarity of *n*_ε_ and of the *n*_*i*_s must be reversed by subtracting their values from the maximum *n*_*i*_.
